# Nitrogen Storage in Rice: Analysis of Physical Quality by Respiration, Weight, and Storage According to Nitrogen Ratio

**DOI:** 10.3390/foods13223530

**Published:** 2024-11-05

**Authors:** Dong Gwan Shin, Jae Woong Han, Jae Hwan Ahn, Hoon Kim

**Affiliations:** 1 Department of Agricultural Engineering, Kongju National University, Yesan 32439, Republic of Korea; sdgs@kfri.re.kr; 2 Food Safety and Distribution Research Group, Korea Food Research Institute, Wanju 55365, Republic of Korea; jhahn@kfri.re.kr; 3 Department of Smartfarm Engineering, Kongju National University, Yesan 32439, Republic of Korea; hanwoong@kongju.ac.kr

**Keywords:** rice storage, nitrogen storage, nitrogen ratio, respiration suppression

## Abstract

Various studies have been conducted to minimize the damage and loss of stored grain. For safe storage, the moisture content must be reduced, or respiration must be suppressed. In this study, grain respiration rates were analyzed under various nitrogen atmospheric conditions, and the quality of stored rice was evaluated. As the nitrogen content of the storage space increases, the respiration rate of the grain decreases accordingly. In this study, the effect of the modified atmospheric nitrogen concentration on reducing respiration was determined. When predicting weight loss due to respiration, low moisture content, and high nitrogen concentration could reduce loss. Quality analysis was performed to compare different respiration rate conditions and showed that lower oxygen concentration and moisture content were associated with safer storage. Our results indicate that changes in atmospheric conditions depending on climate and storage conditions can be considered for the safe storage of harvested rice.

## 1. Introduction

Similar to wheat and legumes, rice is a crop consumed globally and is a staple food in most countries [1,2]. After harvesting, rice is stored for a certain period and is polished before consumption [3]. Although rice is typically stored for less than a year before consumption, governments of countries that consume rice as a staple, such as Korea, Japan, and China, sometimes store unpolished or brown rice for up to five years to bolster reserves.

Moisture content is an important quality factor in storage, and storing rice at a low moisture content helps protect it from physical and chemical changes [4,5,6]. Considering the storage duration, the ideal moisture content is <15% wet basis (%, w.b.) for short-grain varieties and 13%, w.b. for long-grain varieties and is closely related to milling. Specifically, to minimize rice breakage and energy generation, an adequate moisture content must be maintained during the milling process.

Even if rice is stored at an appropriate moisture content, it undergoes respiration through metabolic processes [7,8,9]. Respiration is the most important storage indicator and is closely related to the deterioration of product quality and mass loss [10,11,12]. In particular, a high storage temperature is associated with an increased respiration rate and accelerated deterioration. The degradation of 1 g of dry matter of rice through respiration produces 1.07 g of CO_2_, 0.6 g of water, and 15.4 kJ of energy [13]. Thus, based on Fick’s second law, given a value for CO_2_ diffusivity, a CO_2_ generation model can be used to predict dry mass loss occurring during storage [14,15].

Rice is typically stored in silos at an ambient temperature. When the temperature rises in summer, it is stored at lower temperatures of approximately 15 °C, using a grain cooler or refrigerator [16]. A modified atmosphere (MA) and airtight storage are sometimes employed in subtropical areas; however, installation and maintenance of the required equipment are costly.

Low-temperature storage is reported to be the most effective method for maintaining quality [17], but when the external temperature is high, condensation in the storage facilities becomes a concern. The prevention of condensation requires insulation, which is very expensive. Electricity consumption and carbon emissions are also inevitable side effects of cooling device operations.

The air is mostly composed of three main types of gases: 79% N_2_, 20–21% O_2_, and 0.04% CO_2_. MA storage involves changing the composition of these three gases in the air for effective storage [18,19]. This method is relatively inexpensive and can effectively maintain quality and delay deterioration by slowing respiration [10,20]. MA storage is effective in preventing pest infestation and reducing pest activity. While previous MA storage studies have extensively explored the use of CO_2_ [21,22,23,24,25], there is a lack of research on the effects of nitrogen storage on respiration and physical characteristics.

Nitrogen is the most abundant gas in the air and can be used in a relatively safe manner for MA storage. A higher nitrogen concentration in MA storage results in a lower oxygen concentration. In other words, the respiration rate of rice differs depending on the nitrogen concentration, which is also expected to affect the extent of deterioration. Selecting the appropriate nitrogen concentration for commercial use in MA storage is important, as it can help reduce storage costs. In this study, we aimed to investigate how nitrogen concentration during MA storage affects the respiration and quality characteristics of rice.

## 2. Material and Methods

### 2.1. Materials

The rice used in this experiment was the mid-to-late maturing, short-grain ‘Chucheong’ variety harvested in October from Hwaseong-si, Gyeonggi-do, Republic of Korea. After harvesting, a grain sorter (F2, Ogihara, Japan) was used to filter out empty kernels and foreign materials; the initial moisture content at this point was 25.3%, w.b. The rice was then dried using a laboratory dryer (HSED-M, Hansung, Republic of Korea) at a mean drying temperature of 40 °C (±0.3 °C) at a drying rate of 0.3–0.5%, w.b./h to a moisture content of 15–24%, w.b. (level 4). Foreign material was separated from the dried sample before the experiment using a screen sieve (SF-31, Yamamoto, Japan). The final moisture contents of the samples used in the experiment were 15.7%, 18.2%, 22.4%, and 23.9%, w.b. The moisture content was measured using an oven dryer (OMS100, Thermo Fisher Scientific Inc., Watham, MA, USA) at 10 g, 130 °C, for 24 h under atmospheric pressure [26].

### 2.2. Respiration Experiment

**Figure 1:**
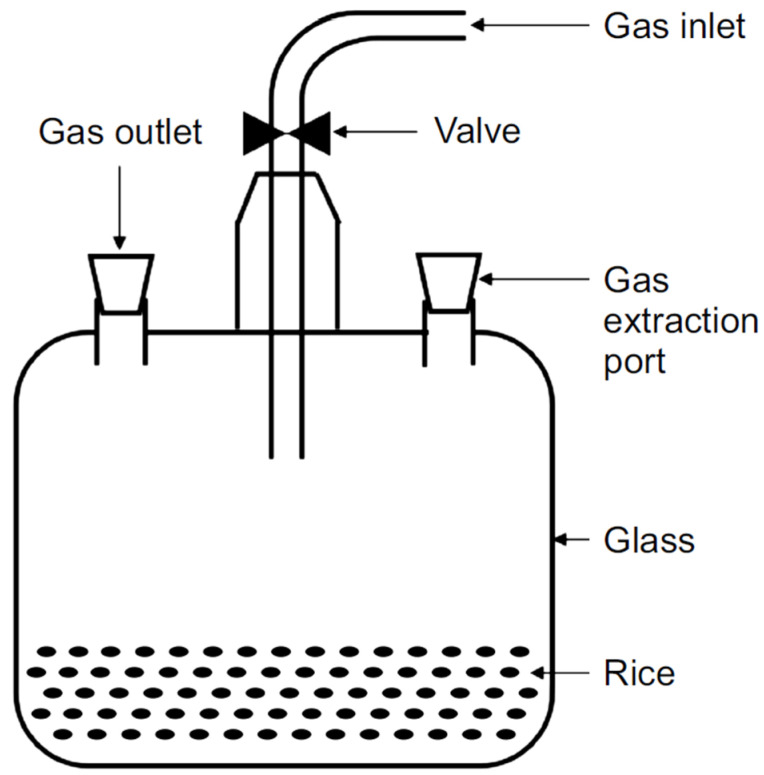
Container for the nitrogen storage experiment.

For respiration measurement, an open-air inlet and outlet valve and an air sample collection port (Figure 1) were mounted on a 1000 mL sealed glass container. Rice weighing 250 g was placed inside a sealed glass container. To assess the differences in respiratory volume, air containing 78%, 85%, 93%, and 100% nitrogen was injected into the glass container through the inlet valve for at least 10 min, after which the outlet valve was opened. The oxygen concentration was divided into four stages, 22%, 15%, 7%, and 0%, through the introduced nitrogen.

Once the nitrogen concentration was set, the glass container was stored in an incubator (BF250LTI, Biofree, Seoul, Republic of Korea) at a constant temperature of 22.0 °C (±0.1 °C). Gas chromatography (GC-14 APT, Shimadzu, Japan) was used to measure the changes in gas composition inside the container during storage. Using a syringe, 0.2 mL of gas was collected from the gas collection port of the container. A CTR-1 (CTR-1, Altech, CA, USA) GC column was used, and the temperature was set at 30 °C. Helium was used as the carrier gas at a flow rate of 50 mL/min. A thermal conductivity detector was used with an injector and a temperature of 60 °C.

### 2.3. Respiration Model and Dry Mass Loss

Although the respiration process in rice is an enzymatic reaction, its characteristics and prevention can be determined based on the stored grain deterioration. The respiration model is an equation based on the respiration rate and volume [27]. The volumes of CO_2_ produced and O_2_ consumed were calculated using Equation (1), and the volume of the container for measuring respiration rate was obtained using the density equation [12,28].

(1) $${\;D}_{g}=\frac{\;C\times V\;}{100\times W}$$

where $D_{g}$ represents the volume of CO_2_ produced or O_2_ consumed (mL/kg dry matter). C is the concentration of O_2_ or CO_2_ (%) measured using GC, V is the volume of the container (mL), and W is the dry weight of the sample (kg).

Using Equation (2), CO_2_ production per unit mass of dry matter was obtained from CO_2_ density. The respiration volume was obtained from its relationship with the measurement time.

(2) $$D=D_{g}\times \rho_{c}$$

where D is the amount of CO_2_ produced per unit mass (mg/kg dry matter), and $\rho_{c}$ is the CO_2_ density (mg/mL).

A model was developed from the values in the equations above, and the Arrhenius equation (Equation (3)) was used in the respiration model [29,30].

(3) $$R=R_{a}\exp\left(-\frac{a}{T}\right)$$

where R is the respiration rate (mg/h kg dry matter). $R_{a}$ is the specific respiration coefficient of the rice (mg/h kg dry matter), a is the specific temperature coefficient of the rice (K), and T is the temperature of the rice (K).

In the Arrhenius equation, $R_{a}$ and a are assumed to be functions of the moisture content. As shown in Equation (4), the rice respiration rate is a function of the moisture content and nitrogen concentration, and the experimental constants were determined by fitting the experimental values using nonlinear regression [31,32,33].

(4) $$R=\left(a+\mathrm{bM}+{\mathrm{cM}}^{2}\right)\exp\left(\frac{d+\mathrm{eM}+{\mathrm{fM}}^{2}}{N}\right)$$

where M is the moisture content (%, dry basis), N is the nitrogen concentration (%), and a,b,c,d,e,f are experimental constants.

Owing to the respiratory activity of rice even after harvesting, as shown in Equation (5), for every 1 g of carbohydrate degraded, 1.47 g of CO_2_, 0.6 g of water, and 3.76 kcal of energy are generated [34].

(5) $$C_{6}H_{12}O_{6}+6O_{2}=6{\mathrm{CO}}_{2}+6H_{2}O+3.76\;\mathrm{kcal}\;$$

Thus, the loss of dry mass during storage can be calculated from the amount of CO_2_ and energy produced. When 1 kcal of energy is generated per kg dry mass, 0.26586 g (0.026598% of the dry mass) of carbohydrates is degraded. When 1 g of CO_2_ is produced per kg dry mass, 0.68182 g (0.068182% of the dry mass) of carbohydrate is degraded. The rate of dry mass loss can be expressed as shown in the equation below. The rate of dry mass loss owing to respiration during storage was calculated based on Equations (4) and (6):

(6) $$\mathrm{DML}=\frac{0.068182\times R}{1000}\;$$

where DML is the rate of dry mass loss over time (%), and R is the respiration rate (CO_2_ mg/h kg dry matter).

### 2.4. Quality During Storage

Using the same method as that used for the respiration experiment, 5000 mL glass containers containing 2.5 kg of rice were prepared with nitrogen concentrations of 78%, 85%, 93%, and 100% and stored for 300 days in an incubator at a constant temperature of 22.0 °C (±0.1 °C). Throughout the storage period, samples were collected to measure the moisture content, germination rate, fat acidity, total bacterial count, and mold count.

The moisture content was measured by the oven-drying method using 10 g, 130 °C, for 24 h under atmospheric pressure. The located rice was selected and prepared, and a germination paper was prepared by placing 100 rice seeds on a moisture-added germination paper. The germination temperature was maintained at 25 °C for seven days, and the number of germinated grains out of 100 was measured. The value of fat acidity was obtained using the AACC method 02-01.02 [35].

Ten grams of rice was collected and placed in a sterilized bag with 90 mL of sterilized water to measure the change in total bacterial and mold counts. The mixture was then homogenized, and 0.1 mL of the sample was inoculated onto plate count agar medium. After incubation for 48 h at 25 °C, the number of colonies was counted and expressed in terms of colony-forming units per g (CFU/g) [36,37].

### 2.5. Statistical Analysis

Statistical analysis was performed using nonlinear regression analysis in SAS (SAS ver. 9.4, SAS Institute, Cary, NC, USA) based on the respiration rate measurements for different nitrogen concentrations and moisture contents, and a respiration model was developed.

## 3. Results and Discussion

### 3.1. Gas Composition and Change in Respiration Volume

**Figure 2:**
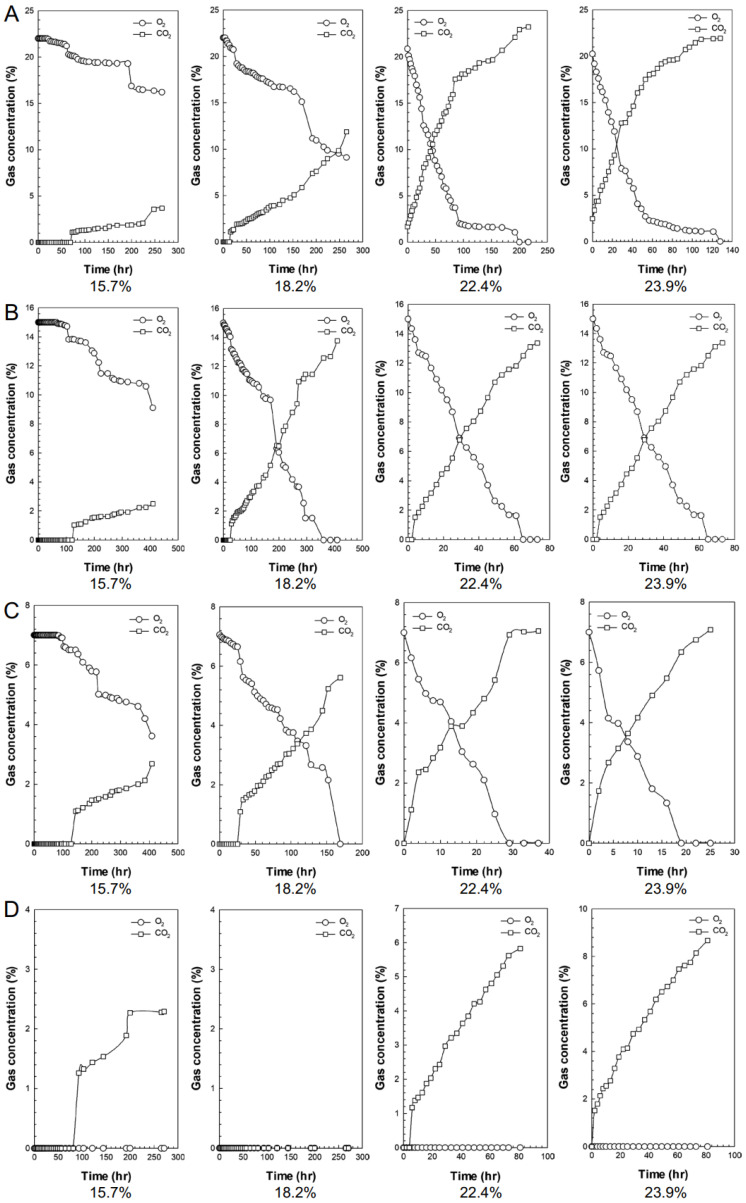
Changes in gas composition according to the nitrogen concentration and moisture content during rice storage ((**A**) = N_2_ 78%, (**B**) = N_2_ 85%, (**C**) = N_2_ 93%, (**D**) = N_2_ 100%).

Grains consume oxygen and produce carbon dioxide through respiration [10,38]. Increasing the nitrogen concentration when the sample’s moisture content is low leads to reduced CO_2_ production. The time required for CO_2_ to increase to 2% due to respiration at 78% nitrogen was 200, 30, 2, and 1 h at moisture contents of 15.7%, 18.2%, 22.4%, and 23.9%, w.b., respectively. When the moisture content was 15.7%, w.b., the time taken for CO_2_ to increase to 2% at the nitrogen concentrations of 78, 85%, and 93% was 200, 300, and 360 h, respectively. At the 100% nitrogen concentration, no CO_2_ was produced. When the moisture content was 18.2%, w.b., the time taken for CO_2_ to increase to 2% at the nitrogen concentrations of 78, 85%, and 93% was 30, 55, and 60 h, respectively. Minimal differences between the different nitrogen concentrations were found at the high moisture content of 22.4% and 23.9%, w.b. The increase in nitrogen concentration was associated with slow CO_2_ production, indicating the inhibition of respiratory activity. However, this effect was mitigated by increasing the moisture content. At all nitrogen concentrations of 22.4% and 23.9%, w.b. CO_2_ production increased rapidly. The low moisture content of grains inhibited respiratory activity, thereby reducing oxygen consumption [10,38] (Figure 2).

**Figure 3:**
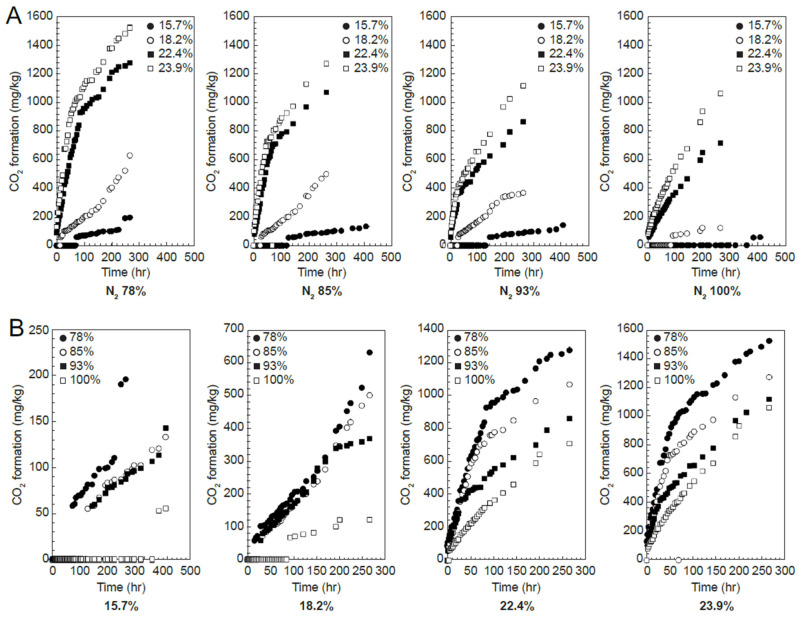
Changes in respiration (CO_2_ generation) during rice storage according to moisture content (**A**): circles = 15.7%, empty circles = 18.2%, squares = 22.4%, empty squares = 23.9%, w.b.) and nitrogen composition (**B**): circles = N_2_ 78%, empty circles = N_2_ 85%, squares = N_2_ 93%, empty squares = N_2_ 100%).

In the CO_2_ generation graph, the CO_2_ generation amount increased rapidly under all nitrogen concentration conditions as the storage time elapsed. The increase was low under conditions below 20%, w.b. moisture content, especially at 15.4%, w.b. (Figure 3). The increase in CO_2_ leads to the generation of moisture and the decrease in the weight of the buildings due to the deterioration of the grains.

### 3.2. Respiration Model and DML

**Figure 4:**
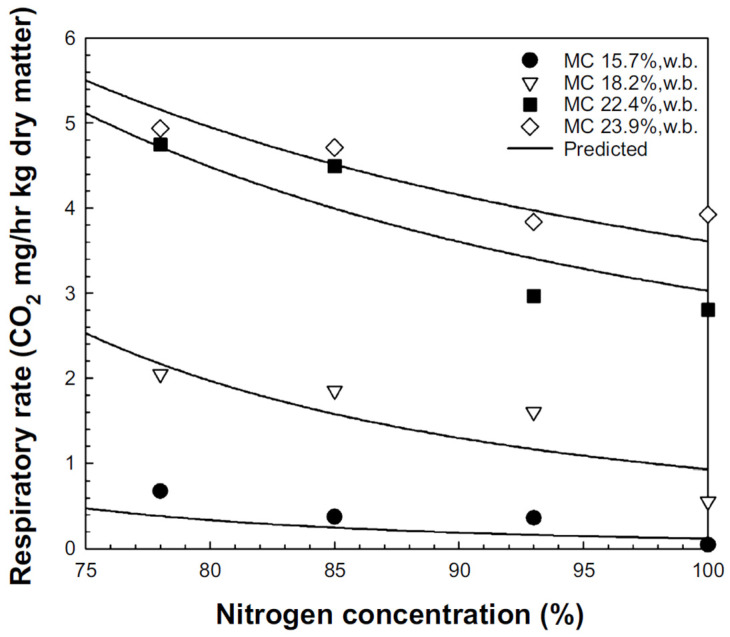
Comparison of the actual and predicted values for the rice respiration rate based on nitrogen concentration and moisture content (circles = 15.7%, down triangles = 18.2%, squares = 22.4%, diamonds = 23.9%, w.b.).

By 2050, an estimated 70% increase in food production will be required, and high levels of grain loss, potentially exceeding 20%, present a central challenge for the rice industry [39,40,41]. DML occurs due to oxygen exposure, and predicting this can provide valuable information for rice storage facilities and distributors [42]. Using the respiration volume data from the four moisture content and nitrogen concentration levels, the experimental constants in Equation (1) were determined using nonlinear regression (Table 1). The measured values for each condition (moisture content and nitrogen concentration) were compared with the predicted values based on Equation (1) and Table 1, as shown in Figure 3. Table 2 lists the RMSE, SSE, and r^2^ values for the measured and predicted values at each nitrogen concentration. As shown in Table 2, at nitrogen concentrations in the range of 78–100%, the RMSE between the measured and predicted values was 0.097–0.166, SSE was 0.025–0.121, and r^2^ was 0.975–0.995, indicating a good fit. Compared with the other conditions, the measured values at a moisture content of 22.4%, w.b. and a nitrogen concentration of 93% showed a greater discrepancy with the predicted values. Nevertheless, as shown in Table 2, the overall fit was sufficient for model acceptance (Figure 4).

**Figure 5:**
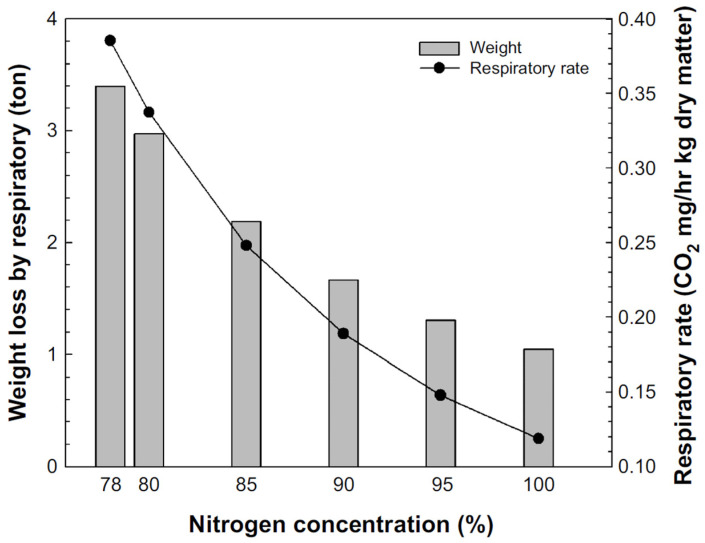
Respiration rate and weight loss due to respiration according to nitrogen concentration during storage (3000 kg).

Figure 5 shows the respiration rate and mass loss at various nitrogen concentrations calculated from Equations (4) and (6) when 3000 tons of rice was fed at a moisture content of 15.7%, w.b. and stored for 180 days. Under normal conditions (78% nitrogen), the respiration rate was calculated to be 0.386, and the mass loss was 3.4 tons. At nitrogen concentrations of 80%, 85%, 90%, 95%, and 100%, the respective mass losses were calculated to be 3.0, 2.2, 1.7, 1.3, and 1.0 tons (respiration rate: 0.337, 0.248, 0.189, and 0.148); the mass loss was reduced by 14.3%, 55.4%, 104.1%, 160.5%, and 224.5%, respectively, compared to normal conditions (Figure 5). Rice is stored in silos and warehouses for both short and long terms, and minimizing weight loss benefits food security and the environment.

### 3.3. Storage Characteristics

#### 3.3.1. Moisture Content

**Figure 6:**
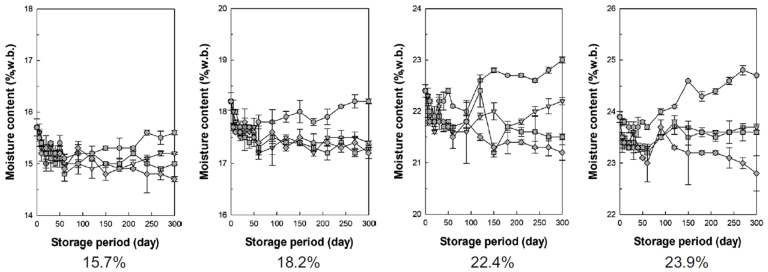
Changes in moisture content during storage according to the nitrogen concentration (circles = N_2_ 78%, triangles = N_2_ 85%, squares = N_2_ 93%, diamonds = N_2_ 100%; the numbers below the graph represent the moisture content on a wet basis).

Figure 6 shows the changes in moisture content over time when rice with four moisture content levels (15.7%, 18.2%, 22.4%, and 23.9%, w.b.) was stored at different nitrogen concentrations. In grains, moisture content is a major factor affecting storage quality, making it important to check for changes in moisture content during storage [6,43] (Figure 6).

When the nitrogen concentration was 78%, the lowest moisture content (15.7%, w.b.) exhibited almost no change during storage. In contrast, when the initial moisture content was 18.2%, 22.4%, or 23.9%, w.b., the moisture content tended to increase over time. In particular, the moisture content increased significantly when the initial moisture content was high. At 85%, 93%, and 100% nitrogen, there was almost no change or a slight decrease in the moisture content. When nitrogen concentration was low and moisture content was high, respiration increased sharply, as shown in Figure 4. According to Equation (5), this increase in respiration is predicted to have also raised the heat and moisture content.

#### 3.3.2. Germination Rate

**Figure 7:**
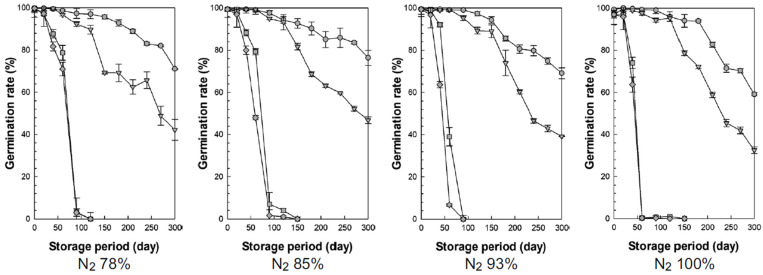
Changes in germination rate during storage according to moisture content (circles = 15.7%, triangles = 18.2%, squares = 22.4%, diamonds = 23.9%, w.b.).

Figure 7 shows the changes in germination rate over time when rice with four moisture content levels (15.7%, 18.2%, 22.4%, and 23.9%, w.b.) was stored under different nitrogen concentrations (Figure 7).

Germination rate, an indicator that affects the seed vigor and taste of rice [44], generally decreases with increasing moisture content, temperature, and extended storage duration [45]. Germination rates tended to decrease as the storage period increased, with higher moisture levels accelerating this decline [46,47,48].

Higher moisture content and longer storage duration were associated with lower germination rates, but the effects of moisture content on the germination rate differed depending on the nitrogen concentration. Specifically, at high moisture contents (22.4% and 23.9%, w.b.), the germination rate declined as the nitrogen concentration increased. At low moisture contents (15.7% and 18.2%, w.b.), the germination rate decreased less quickly or only slightly as the nitrogen concentration increased.

Tahir et al. (2023) conducted general and MA storage at 10% and 14%, w.b. for six months and reported that germination rates were lower in the general storage than in the MA storage [49]. However, Rani et al. (2013) studied germination rates in pinto beans at temperature and moisture content levels that align with the conditions of our experiment [50]. As the storage period increased, germination rates declined. At a temperature close to our experimental condition of 20 °C, a moisture content of 18%, w.b. maintained germination rates above 68%, while moisture levels above 18%, w.b. saw germination rates drop to around 40%. Zhao et al. (2024) reported that low temperatures and nitrogen storage are effective in delaying deterioration and reducing catalase activity, with enhanced effectiveness when used in combination. They also observed that low-temperature storage increased amygdalin levels, contributing to seed viability, although this effect was less pronounced with nitrogen storage alone [19].

In our germination rate experiment, the effect of nitrogen was minimal, while moisture content had a significant impact on germination rates.

#### 3.3.3. Fat Acidity

**Figure 8:**
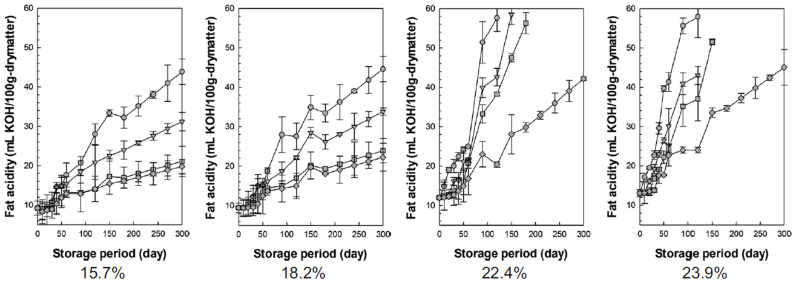
Changes in fat acidity value during storage according to the nitrogen concentration (circles = N_2_ 78%, triangles = N_2_ 85%, squares = N_2_ 93%, diamonds = N_2_ 100%; the numbers below the graph represent the moisture content of wet basis).

Figure 8 shows the changes in fat acidity values over time when rice with four moisture content levels (15.7%, 18.2%, 22.4%, and 23.9%, w.b.) was stored at different nitrogen concentrations (Figure 8). The fat acidity value is a measure of lipid rancidity that increases during storage. Liu et al. (2017) reported that the fat acidity values of all brown rice samples gradually increased over the storage period at 15, 25, and 35 °C. [51,52]. Immediately after harvest, the fat acidity value of fresh rice is approximately 10 mL KOH/100 g dry matter, and rice is considered spoiled when the fat acidity value exceeds 20–25 mL KOH/100 g dry matter [51,53,54]. In other words, the fat acidity value is an important indicator of the shelf-life of rice.

As shown in Figure 8, a higher moisture content and longer storage duration were associated with a trend toward rapidly increasing fat acidity values. At high moisture contents (22.4% and 23.9%, w.b.), the time required for the fat acidity values to reach 20 mL KOH/100 g dry matter was 25, 40, 60, and 65 days at nitrogen concentrations of 78%, 85%, 93%, and 100%, respectively. At low moisture contents (15.7% and 18.2%, w.b.), the time required for the fat acidity value to reach 20 mL KOH/100 g dry matter was 75, 110, 220, and 300 days at nitrogen concentrations of 78%, 85%, 93%, and 100%, respectively. Thus, if a moisture content of 16%, w.b. or less and a nitrogen concentration of 93% or more are maintained, rice can be stored stably for approximately 300 days without a considerable increase in the fat acidity value. Chenling et al. (2022) also reported that fat acidity values increase more slowly in nitrogen storage compared to general storage [55].

#### 3.3.4. Total Bacterial and Mold Counts

**Figure 9:**
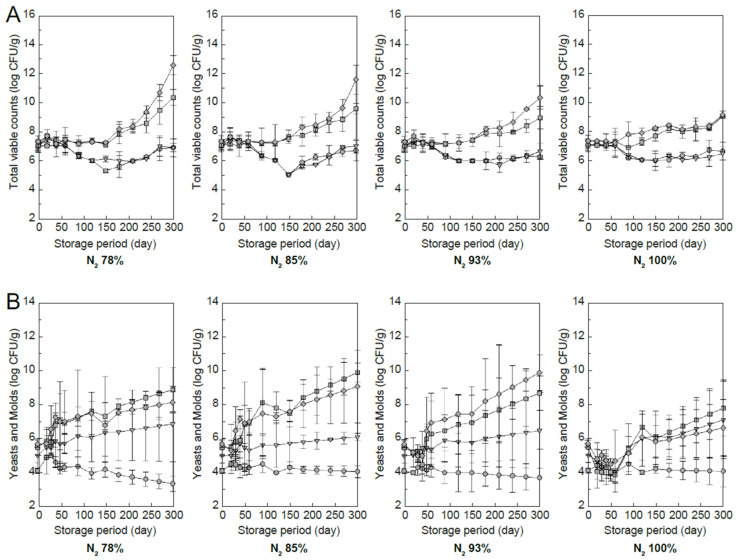
Changes in total bacterial counts according to moisture content (**A**). Changes in mold counts according to moisture content (**B**) (circles = 15.7%, triangles = 18.2%, squares = 22.4%, diamonds = 23.9%, w.b.).

Microbial activity during rice storage significantly deteriorates quality and is a major safety issue owing to mold growth and toxin accumulation [56]. To inhibit this, physical, chemical, and biological methods are being studied [57]. Figure 9 shows the changes in the total bacterial and mold counts over time when rice with four moisture content levels (15.7%, 18.2%, 22.4%, and 23.9%, w.b.) was stored at different nitrogen concentrations (Figure 9).

The total bacterial count tended to increase significantly at high moisture contents (22.4% and 23.9%, w.b.); however, there was almost no change at low moisture contents. As the nitrogen concentration increased, the increase in the total bacterial count was hampered. The mold tended to increase at moisture contents other than 15.7%, w.b. At a nitrogen concentration of 100%, the increase in the mold was lower than that at the other concentrations.

These findings indicate that the total bacterial count and mold growth can be sufficiently controlled if a low moisture content is maintained during storage. However, our experiment was conducted at a temperature of 22 °C, making it difficult to sufficiently control microbial activity at higher storage temperatures, even if the moisture content was kept low. Mold is generally present on the surface of grains and within the seed coat. Under most climatic conditions, some of these molds are known to produce secondary metabolites, which are influenced by two primary factors: temperature and moisture content [56]. At higher storage temperatures, it may be possible to control microbial activity by managing moisture content and using an appropriate nitrogen concentration.

## 4. Conclusions

In this study, nitrogen was used to minimize weight loss by controlling the respiratory rate of rice and to safely store rice. We investigated how changes in nitrogen concentration affect respiration rates and quality characteristics during storage, such as moisture content, germination rate, fat acidity, and microbial activity.

Grains are greatly affected by temperature and moisture content during storage. The most important factors for germination rate are temperature and moisture content, with a sharp decline observed at moisture levels above 20%, w.b. When the fat acidity value was based on 20–25 mL KOH/100 g of dry matter, an increase in fat acidity value was inhibited only when the nitrogen concentration was 93% or higher and the moisture content was stored at 16%, w.b. or below. Mold showed a tendency to increase at moisture contents above 16%, w.b., while total bacterial counts increased above 19%, w.b. Both bacterial counts and mold growth were slower at nitrogen concentrations of 93% or higher. However, maintaining a low moisture content during storage proved to be even more effective. Based on the developed respiration models, if 3000 tons of rice with a moisture content of 15.7%, w.b. is stored for 180 days at a nitrogen concentration of 78%, the respiration rate will be 0.386 and the mass loss will be 3.4 tons. In comparison, at nitrogen concentrations of 85%, 90%, 95%, and 100%, with other conditions constant, the mass loss will decrease by 55.4%, 104.1%, 160.5%, and 224.5%, respectively.

Respiration is highly correlated with grain weight and quality, and the most effective way to inhibit respiration during storage is to sufficiently reduce temperature through cooling. However, energy use during the cooling process increases storage costs and carbon emissions. In this study, we demonstrated that the use of nitrogen during rice storage effectively suppressed the respiration rate, thereby reducing quality deterioration and mass loss.

## Figures and Tables

**Table 1:** The experimental constants used in the model.

a	b	c	d	e	f
2.8337	−0.2895	0.00738	−1440.4	74.0155	−1.0245

**Table 2:** A comparison between the measured and predicted values for the rice respiration rate at varying nitrogen concentrations.

	RMSE	SSE	r^2^
78	0.097	0.041	0.995
85	0.154	0.025	0.996
93	0.166	0.121	0.975
100	0.134	0.076	0.982

## Data Availability

The original contributions presented in the study are included in the article, further inquiries can be directed to the corresponding author.

## References

[1] Hu X., Fang C., Zhang W., Lu L., Guo Z., Li S., Chen M. (2023). Change in volatiles, soluble sugars and fatty acids of glutinous rice, japonica rice and indica rice during storage. LWT.

[2] Wang Y., Zheng Y., Zhou R., Ma M. (2022). Kinetic studies on soluble sugar profile in rice during storage: Derivation using the Laplace transform. Innov. Food Sci. Emerg. Technol..

[3] Choi S., Seo H.S., Lee K.R., Lee S., Lee J., Lee J. (2019). Effect of milling and long-term storage on volatiles of black rice (*Oryza sativa* L.) determined by headspace solid-phase microextraction with gas chromatography-mass spectrometry. Food Chem..

[4] Afzal I., Bakhtavar M.A., Ishfaq M., Sagheer M., Baributsa D. (2017). Maintaining dryness during storage contributes to higher maize seed quality. J. Stored Prod. Res..

[5] Bartosik R., Urcola H., Cardoso L., Maciel G., Busato P. (2023). Silo-bag system for storage of grains, seeds and by-products: A review and research agenda. J. Stored Prod. Res..

[6] Ziegler V., Paraginski R.T., Ferreira C.D. (2021). Grain storage systems and effects of moisture, temperature and time on grain quality—A review. J. Stored Prod. Res..

[7] Kuyu C.G., Tola Y.B., Mohammed A., Mengesh A., Mpagalile J.J. (2022). Evaluation of different grain storage technologies against storage insect pests over an extended storage time. J. Stored Prod. Res..

[8] Timm N.d.S., Coradi P.C., Cañizares L., Jappe S.N., Ferreira C.D., Lutz É. (2023). Effects of the storage temperature and time of corn from the center and extremities of corncob on quality parameters. J. Cereal Sci..

[9] Ubhi G.S., Sadaka S. (2015). Temporal valuation of corn respiration rates using pressure sensors. J. Stored Prod. Res..

[10] Wang R., Zhang L., Lu Q. (2018). Exploration of mechanisms for internal deterioration of wheat seeds in postharvest storage and nitrogen atmosphere control for properties protection. Crop Sci..

[11] Salazar N., Caladcad J.A., Villeta R. (2024). Predictive modelling on the effects of the critical parameters in grain storage systems: A case study in the Philippines. J. Stored Prod. Res..

[12] Barreto A.A., Abalone R., Gastón A., Bartosik R. (2013). Analysis of storage conditions of a wheat silo-bag for different weather conditions by computer simulation. Biosyst. Eng..

[13] Huang H., Danao M.C., Rausch K.D., Singh V. (2013). Diffusion and production of carbon dioxide in bulk corn at various temperatures and moisture contents. J. Stored Prod. Res..

[14] Müller A., Nunes M.T., Maldaner V., Coradi P.C., de Moraes R.S., Martens S., Leal A.F., Pereira V.F., Marin C.K. (2022). Rice drying, storage and processing: Effects of post-harvest operations on grain quality. Rice Sci..

[15] Thompson T.L. (1972). Temporary storage of high-moisture shelled corn using continuous aeration. Trans. ASAE.

[16] Da Silva W.S.V., Vanier N.L., Ziegler V., de Oliveira M., Guerra Dias A.R.G., Elias M.C. (2014). Effects of using Eolic exhausters as a complement to conventional aeration on the quality of rice stored in metal silos. J. Stored Prod. Res..

[17] Xiao Y., Xie L., Li Y., Li C., Yu Y., Hu J., Li G. (2024). Impact of low temperature on the chemical profile of sweet corn kernels during post-harvest storage. Food Chem..

[18] Sun S., Li B., Yang T., Luo F., Zhao J., Cao J., Lin Q. (2019). Preservation mechanism of high concentration carbon dioxide controlled atmosphere for paddy rice storage based on quality analyses and molecular modeling tools. J. Cereal Sci..

[19] Zhao Y., Li Y., Gong Z., Liu X., Lv H., Zhao Y. (2024). Changes in Quality Characteristics and Metabolite Composition of Low-Temperature and Nitrogen-Modified Atmosphere in Indica Rice during Storage. Foods.

[20] Shejbal J. (1979). Preservation of cereal grains in nitrogen atmospheres. Resour. Recovery Conserv..

[21] Hashem M.Y., Ahmed A.A., Ahmed S.S., Khalil S.S., Mahmoud Y.A. (2016). Comparative susceptibility of Corcyra cephalonica (Lepidoptera: Pyralidae) eggs to carbon dioxide and nitrogen at different temperatures. J. Stored Prod. Res..

[22] Navarro S. (2012). The use of modified and controlled atmospheres for the disinfestation of stored products. J. Pest Sci..

[23] Neethirajan S., Freund M.S., Jayas D.S., Shafai C., Thomson D.J., White N.D.G. (2010). Development of carbon dioxide (CO_2_) sensor for grain quality monitoring. Biosyst. Eng..

[24] Taher H.I., Urcola H.A., Cendoya M.G., Bartosik R.E. (2019). Predicting soybean losses using carbon dioxide monitoring during storage in silo bags. J. Stored Prod. Res..

[25] Haritos V.S., Damcevski K.A., Dojchinov G. (2006). Improved efficacy of ethyl formate against stored grain insects by combination with carbon dioxide in a ‘dynamic’application. Pest Manag. Sci. Former. Pestic. Sci..

[26] Grigg B.C., Siebenmorgen T.J. (2015). Impacts of kernel thickness and associated physical properties on milling yields of long-grain rice. Appl. Eng. Agric..

[27] Maciel G., de la Torre D.A., Cardoso L.M., Cendoya M.G., Wagner J.R., Bartosik R.E. (2020). Determination of safe storage moisture content of soybean expeller by means of sorption isotherms and product respiration. J. Stored Prod. Res..

[28] Marcos Valle F.J., Gastón A., Abalone R.M., de la Torre D.A., Castellari C.C., Bartosik R.E. (2021). Study and modelling the respiration of corn seeds (*Zea mays* L.) during hermetic storage. Biosyst. Eng..

[29] Ho P.L., Tran D.T., Hertog M.L.A.T.M., Nicolaï B.M. (2020). Modelling respiration rate of dragon fruit as a function of gas composition and temperature. Sci. Hortic..

[30] Nishiyama M., Kleijn S., Aquilanti V., Kasai T. (2009). Temperature dependence of respiration rates of leaves, 18O-experiments and super-Arrhenius kinetics. Chem. Phys. Lett..

[31] Benítez S., Chiumenti M., Sepulcre F., Achaerandio I., Pujolá M. (2012). Modeling the effect of storage temperature on the respiration rate and texture of fresh cut pineapple. J. Food. Eng..

[32] Mundim K.C., Baraldi S., Machado H.G., Vieira F.M.C. (2020). Temperature coefficient (Q10) and its applications in biological systems: Beyond the Arrhenius theory. Ecol. Model..

[33] Waghmare R.B., Mahajan P.V., Annapure U.S. (2013). Modelling the effect of time and temperature on respiration rate of selected fresh-cut produce. Postharvest Biol. Technol..

[34] Raudienė E., Rušinskas D., Balčiūnas G., Juodeikienė G., Gailius D. (2017). Carbon dioxide respiration rates in wheat at various temperatures and moisture contents. Mapan.

[35] AACC (1999). AACC Method 02-01.02 Fat Acidity-General Method. AACC Approved Methods Analysis.

[36] Jacob A., Sudagar I.P., Pandiselvam R., Rajkumar P., Rajavel M. (2023). Effect of packaging materials and storage temperature on the physicochemical and microbial properties of ultrasonicated mature coconut water during storage. Food Control.

[37] Shi J., Zhang T., Geng S., Liang F., Wang T. (2021). Effect of accumulated temperature on flavour and microbial diversity of japonica rice during storage. J. Stored Prod. Res..

[38] Taher H., San Martino S., Abadía M.B., Bartosik R.E. (2023). Respiration of barley seeds (*Hordeum vulgare* L.) under different storage conditions. J. Stored Prod. Res..

[39] Kamara M.M., El-Aty S.M.A., Elgamal H.W., Soleiman M.R., Mousa M.K., Ueno T. (2019). Effect of storage temperature on storage efficacy, germination and physical characters of some paddy rice cultivars during different storage periods. J. Fac. Agric. Kyushu Univ..

[40] Dowell F.E., Dowell C.N. (2017). Reducing grain storage losses in developing countries. Qual. Assur. Saf. Crop. Foods.

[41] Kumar D., Kalita P. (2017). Reducing postharvest losses during storage of grain crops to strengthen food security in developing countries. Foods.

[42] Borreani G., Tabacco E., Schmidt R., Holmes B., Muck R. (2018). Silage review: Factors affecting dry matter and quality losses in silages. J. Dairy Sci..

[43] Chidananda K.P., Chelladurai V., Jayas D.S., Alagusundaram K., White N.D.G., Fields P.G. (2014). Respiration of pulses stored under different storage conditions. J. Stored Prod. Res..

[44] Kim D.S., Kim Q.W., Kim H., Kim H.J. (2022). Changes in the chemical, physical, and sensory properties of rice according to its germination rate. Food Chem..

[45] Ghosh M., Gorain J., Pal A.K., Saha B., Chakraborti P., Avinash B., Pyngrope D.M., Banerjee S., Sarkar S. (2021). Study of seed morphology and influence of ageing and storage conditions on germination and seedling vigour of non-Basmati aromatic rice. J. Stored Prod. Res..

[46] Nithya U., Chelladurai V., Jayas D.S., White N.D.G. (2011). Safe storage guidelines for durum wheat. J. Stored Prod. Res..

[47] Karunakaran C., Muir W.E., Jayas D.S., White N.D.G., Abramson D. (2001). Safe storage time of high moisture wheat. J. Stored Prod. Res..

[48] Zhang S.B., Lv Y.Y., Wang Y.L., Jia F., Wang J.S., Hu Y.S. (2017). Physiochemical changes in wheat of different hardnesses during storage. J. Stored Prod. Res..

[49] Tahir A., Afzal I., Khalid E., Razzaq M., Arif M.A.R. (2023). Rice seed longevity in the context of seed moisture contents and hypoxic conditions in the storage environment. Seed Sci. Res..

[50] Rani P.R., Chelladurai V., Jayas D.S., White N.D.G., Kavitha-Abirami C.V. (2013). Storage studies on pinto beans under different moisture contents and temperature regimes. J. Stored Prod. Res..

[51] Malegori C., Buratti S., Benedetti S., Oliveri P., Ratti S., Cappa C., Lucisano M. (2020). A modified mid-level data fusion approach on electronic nose and FT-NIR data for evaluating the effect of different storage conditions on rice germ shelf life. Talanta.

[52] Liu K., Li Y., Chen F., Yong F. (2017). Lipid oxidation of brown rice stored at different temperatures. Int. J. Food Sci. Technol..

[53] Qiu Z., Wu F., Hu H., Guo J., Wu C., Wang P., Ling J., Cui Y., Ye J., Fang G. (2024). Deciphering the Microbiological Mechanisms Underlying the Impact of Different Storage Conditions on Rice Grain Quality. Foods.

[54] Salman H., Copeland L. (2007). Effect of storage on fat acidity and pasting characteristics of wheat flour. Cereal Chem..

[55] Qu C., Li W., Yang Q., Xia Y., Lu P., Hu M. (2022). Metabolic mechanism of nitrogen modified atmosphere storage on delaying quality deterioration of rice grains. Food Chem. X.

[56] Mohapatra D., Kumar S., Kotwaliwale N., Singh K.K. (2017). Critical factors responsible for fungi growth in stored food grains and non-chemical approaches for their control. Ind. Crops Prod..

[57] Moses J.A., Jayas D.S., Alagusundaram K. (2015). Climate change and its implications on stored food grains. Agric. Res..

